# Characterising human atherosclerotic carotid plaque tissue composition and morphology using combined spectroscopic and imaging modalities

**DOI:** 10.1186/1475-925X-14-S1-S5

**Published:** 2015-01-09

**Authors:** Hilary E Barrett, John J Mulvihill, Eoghan M Cunnane, Michael T Walsh

**Affiliations:** 1 Centre for Applied Biomedical Engineering Research (CABER), Department of Mechanical, Aeronautical and Biomedical Engineering and Material Surface Science Institute (MSSI), University of Limerick, Limerick, Ireland

## Abstract

Calcification is a marked pathological component in carotid artery plaque. Studies have suggested that calcification may induce regions of high stress concentrations therefore increasing the potential for rupture. However, the mechanical behaviour of the plaque under the influence of calcification is not fully understood. A method of accurately characterising the calcification coupled with the associated mechanical plaque properties is needed to better understand the impact of calcification on the mechanical behaviour of the plaque during minimally invasive treatments. This study proposes a comparison of biochemical and structural characterisation methods of the calcification in carotid plaque specimens to identify plaque mechanical behaviour.

Biochemical analysis, by Fourier Transform Infrared (FTIR) spectroscopy, was used to identify the key components, including calcification, in each plaque sample. However, FTIR has a finite penetration depth which may limit the accuracy of the calcification measurement. Therefore, this FTIR analysis was coupled with the identification of the calcification inclusions located internally in the plaque specimen using micro x-ray computed tomography (μX-CT) which measures the calcification volume fraction (CVF) to total tissue content. The tissue characterisation processes were then applied to the mechanical material plaque properties acquired from experimental circumferential loading of human carotid plaque specimen for comparison of the methods.

FTIR characterised the degree of plaque progression by identifying the functional groups associated with lipid, collagen and calcification in each specimen. This identified a negative relationship between stiffness and 'lipid to collagen' and 'calcification to collagen' ratios. However, μX-CT results suggest that CVF measurements relate to overall mechanical stiffness, while peak circumferential strength values may be dependent on specific calcification geometries. This study demonstrates the need to fully characterise the calcification structure of the plaque tissue and that a combination of FTIR and μX-CT provides the necessary information to fully understand the mechanical behaviour of the plaque tissue.

## Introduction

Symptomatic carotid artery embolisation, acute occlusion and distal propagation of thrombus are a consequence of an atherosclerotic plaque rupture during clinical endovascular intervention [1]. There is clinical and experimental evidence suggesting that rupture locations in carotid artery plaque tissue can be associated with regions of high stress concentrations induced by calcification inclusions. Clinical intervention using intravascular angioplasty is associated with plaque fracture and dissection occurring adjacent to the calcification inclusions [2]. Stenting and angioplasty procedures have also had poor success outcomes in severely calcified vessels due to resistance to high inflation pressures as well as, inadequate and asymmetrical stent expansion [3].

The screening of high risk patients and accurate computational modelling of associated procedural risk for patient-specific plaque types requires non-invasive imaging that permits accurate structural characterisation along with mechanical stiffness and threshold strength values acquired from experimental mechanical testing. One study that attempted to characterise aortic plaque samples tested *in vitro *calcified aortic tissue strips, imaged by x-ray. These plaques were found to have a lower breaking stress and a significantly lower stretch ratio under uniaxial circumferential loading which suggests that calcification reduces the breaking strength of the artery wall [4]. Conversely in another study, on the basis of Fourier Transform Infrared (FTIR) classification using the ratio of calcification to lipid at a threshold level of one in human carotid plaques, predominantly calcified specimens exhibited a significantly higher Cauchy stress and significantly lower stretch ratio in whole plaque circumferential loading [5] therefore suggesting that calcification causes a stiffening effect where plaques will provide more resistance to deformation until the stress exceeds the strength and rupture occurs. Calcification inclusions may immobilise the surrounding plaque tissue causing localised high stress concentrations in the calcified regions where tissue breakdown may occur under excessive loads [4]. However, the mechanical behaviour of the plaque under the influence of calcification is not fully understood and there is a clear need to use more accurate methods of measuring calcification in order to better understand the effect that calcification may have on mechanical behaviour of plaque tissue.

A wide range of imaging techniques has been employed to determine plaque morphology and calcification detail. These include *in vitro *techniques such as x-ray, histology, scanning electron microscopy (SEM), FTIR and *in vivo *techniques such as magnetic resonance imaging (MRI), ultrasound (US) and computed tomography (CT). The *in vitro *based modalities require blind serial sectioning which loses key morphological data and represent a 2D cross-section of a 3D plane which may underestimate the true diameter of the calcification due to the absence of volumetric measures [6]. Moreover, *in vivo *calcification measurement methods, including the degree of calcification, percentage area, volume and density measurements, have led to conflicting conclusions regarding a plaque's biomechanical stability and its association with clinical events. It has been documented previously that FTIR has a finite penetration depth which may limit the accuracy of the FTIR data in characterising calcifications which are located deep within the plaque wall. Therefore, a method of accurately characterising the calcification structures throughout the whole plaque sample coupled with acquiring the mechanical properties is required to better understand the impact of calcification on the mechanical behaviour of plaque including the risk of rupture.

Advancements in imaging resolutions have permitted the reconstruction of 3D volumetric diseased arterial vessels with detail of the internal tissue structure [7]. Furthermore, micro x-ray computed tomography (μX-CT) has permitted patient-specific predictive finite element models that simulate the manipulation of plaque tissue by stent expansion to better encapsulate a realistic evaluation of the tissue injury incurred during the procedure [8]. However, studies are limited in their theoretical predictions as they lack the biological detail and experimental data required to correctly evaluate the tissue injury under certain loading conditions [9].

The overall aim of this study is to demonstrate a method of relating the biochemical and structural calcification characteristics of carotid plaque tissue using FTIR analysis and high resolution μX-CT. However, it is first necessary to compare the improved accuracy of the μX-CT to the FTIR when measuring the amount, size and location of the calcifications within the plaque tissue. This study investigates experimentally derived mechanical properties using a complementary tissue characterisation process on a sample set of six carotid plaque specimens that were previously characterised using FTIR [5]. The previous plaque classification approach that was employed grouped the plaques mechanical response based on a calcification to lipid ratio using a threshold level of one. This led to a degree of overlap between the mechanical stiffness groupings especially when the ratio is close to one. This present study applies a reclassification of a sample set of the plaques along with an additional post-mechanical structural characterisation using µX-CT to the experimentally derived mechanical properties in order to better understand the mechanical behaviour of diseased plaque tissue. Scanning Electron Microscopy-Energy Dispersive X-ray (SEM-EDX) chemical analysis is used to validate the presence of calcification and also to examine the characteristic surface defects in severely advanced plaque types.

## Methods

### Sample acquisition

Carotid plaque specimens were acquired from standard endarterectomy procedures performed in the University of Limerick Hospital Ireland in a manner that conformed to the Declaration of Helsinki and was approved by the hospital's Ethical Research Committee. On the basis of the aforementioned FTIR classification approach a sample set of six carotid plaque samples, which did not conform to the proposed calcification to lipid ratio criteria, were selected for further characterisation in this present study.

### Mechanical testing

Mechanical testing was performed to investigate the circumferential Cauchy stress (MPa) and stretch ratio response of six whole carotid plaque samples using testing parameters that closely replicate the *in vivo *physiological instantaneous systolic pulse. The experiment used five preconditioning cycles, to 10% gauge length, at a displacement rate of 0.1 mm/s to achieve a repeatable stress-stretch response and the tissue was subsequently stretched to complete failure at a displacement rate of 30% of gauge length per second [5]. Cauchy stress and stretch ratio plots were used to quantify the stress induced on the plaque and the deformation response up to the point of ultimate strength.

### Fourier Transform Infrared (FTIR) analysis

**Figure 1:**
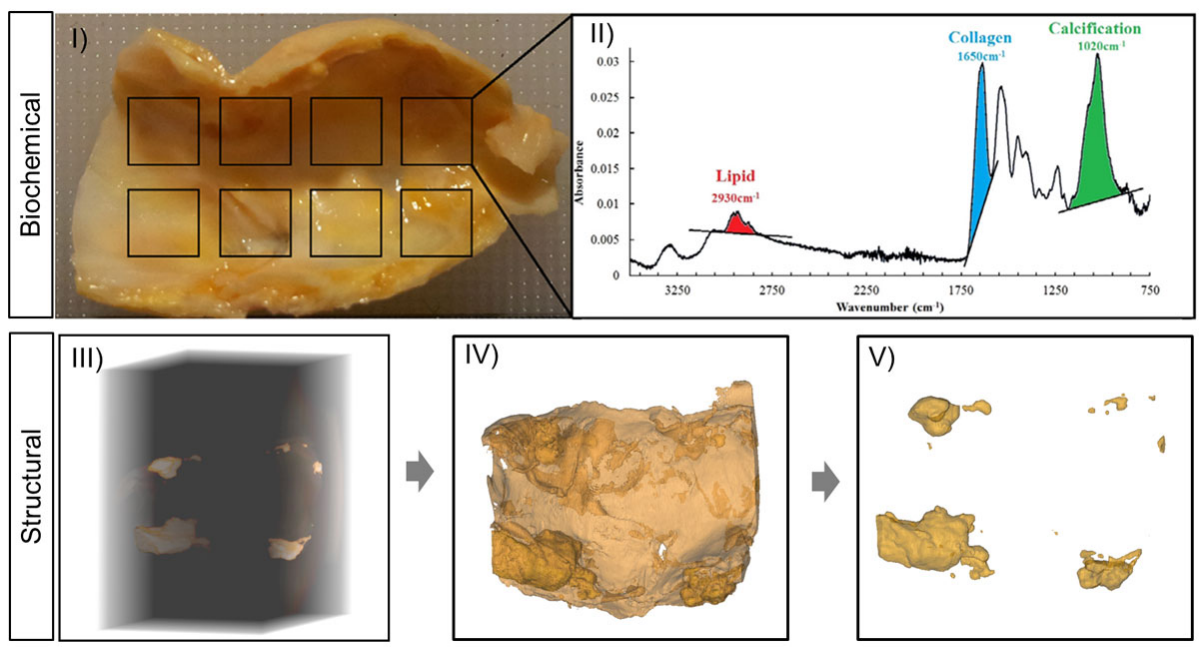
**Tissue characterisation process; biochemical (FTIR) analysis identifying the composition of the carotid plaque specimen at multiple locations (I) acquiring a spectra for each region detailing lipid, collagen and calcification (II)**. Structural characterisation (µX-CT scanning) (III) reconstructed plaque specimen (IV) and calcification segmentation (V).

Probe based spectroscopic Fourier Transform Infrared (FTIR) (Perkin-Elmer Model 1740) was employed to globally characterise the diseased tissue. Samples were analysed in their hydrated state at multiple intimal surface locations. The acquired spectral information was measured through the absorbance in the mid infrared region 4000-700 cm^-1 ^for 16 scans using a spectral resolution of 2 cm^-1^. The tissue sample was then placed on the crystal area plate and the attenuated total reflectance (ATR) probe was positioned over the plaque sample applying an adequate force which does not damage the tissue but ensures full contact. Each spectrum represented the unique chemical composition of lipid, collagen and calcification in a plaque sample relating to chemical compounds absorbed at particular wavenumbers as illustrated in Figure 1. The tissue content was quantified using an absorbance peak-area ratio function in the Spectrum software (Spectrum 100 FTIR). This peak-area measure is directly related to the concentration of each component. The average ratios for lipid to collagen (Lip:Col) and calcification to collagen (Calc:Col) were quantified for each plaque [10].

### μX-CT scanning process

A section from each of the mechanically ruptured plaque samples was extracted and prepared for analysis by μX-CT. The longitudinal and circumferential dimensions of the scanned sections were (12.34 ± 2.18 mm) and (8.85 ± 0.73 mm) respectively. All sections underwent a tissue preservation process involving three stages; fixation, dehydration and drying. This ensured minimal tissue damage under the intense x-ray voltage source. The samples were imaged using Xradia versa XRM 500 (Carl Zeiss X-ray Microscopy Inc.). The imaging process involved the repeated axial scanning of the rotating plaque section. Each plaque specimen was imaged using a 0.4× optical magnification and 2.5s x-ray exposure time. The x-ray source was operated at 50 kV and 81 µA. All tomographic slices were obtained with 15.5 µm pixel resolution producing a voxel volume of (15.5 µm* 15.5 µm* 15.5 µm).

### μX-CT post processing

Three dimensional reconstructions were generated using Xradia XRM reconstructor (version 7.0.2817). The pixel values were rescaled using a standard Hounsfield unit calibration using air, water and hydroxyapatite phantom and the optimum difference in absorbance between the plaque tissue and calcification was achieved using 16-bit grey level images [11]. The reconstructed image slices were exported as a series of tiff images and imported into Mimics Medical imaging processing software, (version 15.0, Materialise, Belgium) for analysis. All slices were density calibrated based on defining a fixed threshold range for the plaque tissue (324-1249GV) and calcification (>1250GV) and a mask was generated for each component (Figure 1). Successful segmentation was achieved due to the high atomic number associated with calcification which strongly attenuates the x-ray emitted by the CT scanner. The lipid component was not segmented as a result of its poor contrast differentiation with plaque tissue. However, the global FTIR analysis adequately identified the lipid concentration within the plaque tissue.

### Quantitative analysis

The individual calcification inclusions were assessed using their three dimensional voxel volume (mm^3^), surface area (mm^2^) and spatial distribution measurements. The ratio of calcification to total plaque volume was calculated for each plaque and also used as an overall calcification measure. The calcification inclusions were then divided into three groups based on geometrical shape and size. The first group are the spherical inclusions defined as any particle less than 300 µm with a sphericity measure close to one (ψ = (π^1/3^(6V_p_)^2/3^)/A_p_) where V_p _and A_p _are volume and surface area measures of the particles [6]. These inclusions are assumed to be spherical which allowed the normalised diameter to be extracted from the measured volumes [11]. The second group, sheet-like structures, were defined as any inclusion that had a thickness (t), width (w) and length (l) measure that conformed to longer than wide and wider than thick aspect ratio (t<<W<<L). The final group consisted of irregularly shaped agglomerated macro-nodes defined as randomly shaped masses of calcification.

### Scanning electron microscopy-energy dispersive X-ray (SEM-EDX) analysis

SEM-EDX chemical analysis was performed on localised calcified regions to validate the μX-CT characterisation and to investigate the ultra-structural surface features. These regions were selected based on the μX-CT analysis. The plaques were re-sectioned and coated in gold (35 mA for 120s) to ensure a sufficient conducting surface using Emitech K550 (Emitech Ltd. U.K.). This analysis produced x-ray spectra, similar to the FTIR spectroscopy spectra, whereby measured relative intensity of x-ray spectral peak is proportional to the mass concentration of the sample elements. The calcium to phosphorus ratio for each inclusion was measured to verify the μX-CT identified inclusions as calcification alternatively known as hydroxyapatite.

## Results

**Figure 2:**
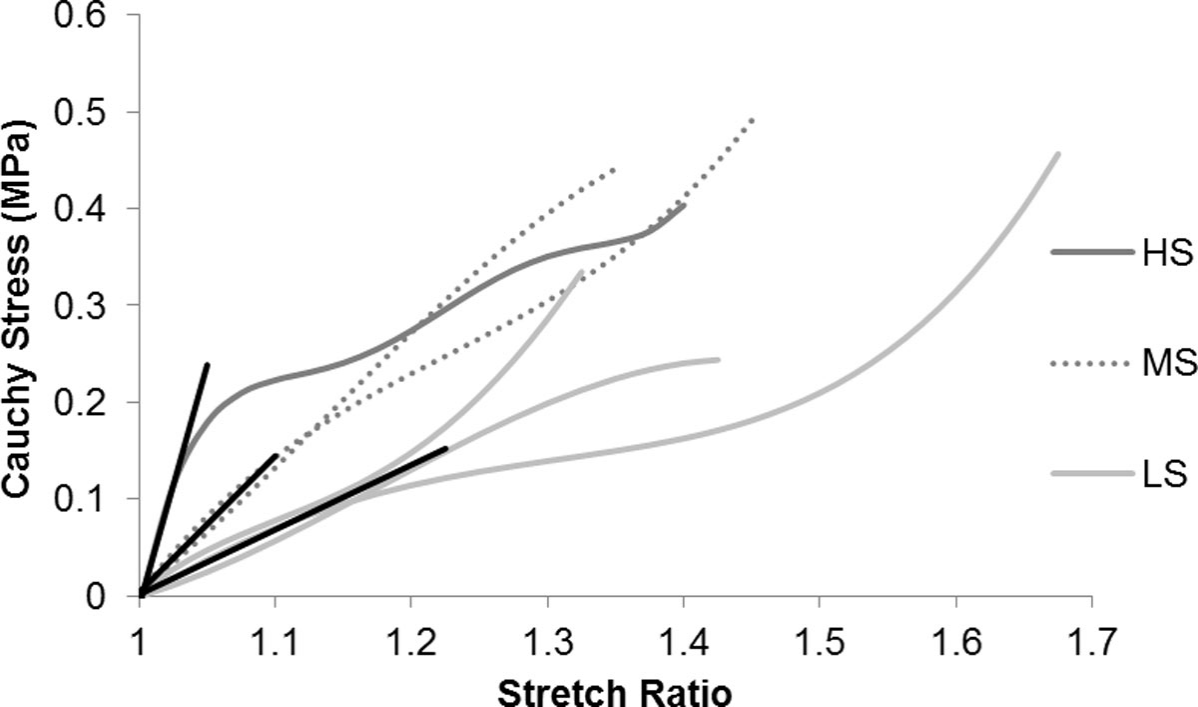
**Cauchy stress and stretch ratio plots of the plaque samples grouped by initial stiffness**. The dark grey represents high stiffness (HS), the dashed line represents medium stiffness (MS) and the light grey represents low stiffness (LS). Note Mechanical data extracted from Mulvihill et al. 2013.

Table 1 summarises the structural µX-CT results for each carotid specimen along with the previously published biochemical and experimental mechanical data [5]. This sample set is tabulated in order of increasing (low to high) initial stiffness (MPa). The Cauchy stress and stretch ratio response to the uniaxial testing of whole human carotid plaque samples in the circumferential loading direction is presented in Figure 2. The heterogeneous morphology of each plaque is demonstrated by the large inter-specimen disparity in mechanical behaviour. Three distinct stiffness levels were exhibited and each plaque was grouped based on their respective level of initial stiffness. The colour of the lines (light grey; grey dashed and dark grey) indicates the three mechanical stiffness levels (LS, low stiffness; MS, medium stiffness and HS, high stiffness) respectively. The stress and stretch data quantify the stress induced on the plaque tissue structure during the large deformation. The mean peak circumferential strength value (± standard deviation) was Cauchy stress 0.40 ± 0.09 MPa and stretch ratio 1.44 ± 0.13.

**Table 1:** Mechanical; initial stiffness and ultimate strength, biochemical; calcification and lipid composition and structural; calcification volume fraction (CVF) and surface area (SA) data for each carotid plaque specimen examined in this study.

	**Mechanical**			**Biochemical**		**Structural**	
			
**Sample**	**Initial stiffness (MPa)**	**Cauchy Stress (MPa)**	**Stretch ratio**	**Calc:col**	**Lip:col**	**CVF**	**Surface Area (mm^2^)**
			
**LS**	0.6	0.24	1.43	0.24	0.67	0.11	15.05
**LS**	0.65	0.45	1.68	0.63	1.55	0.32	31.58
**LS**	0.75	0.33	1.33	0.66	0.97	0.08	9.41
**MS**	1.25	0.44	1.35	0.68	0.41	0.19	10.73
**MS**	1.6	0.49	1.45	0.22	0.36	0.13	9.55
**HS**	3.79	0.40	1.4	0.30	0.20	0.52	130.85

**Figure 3:**
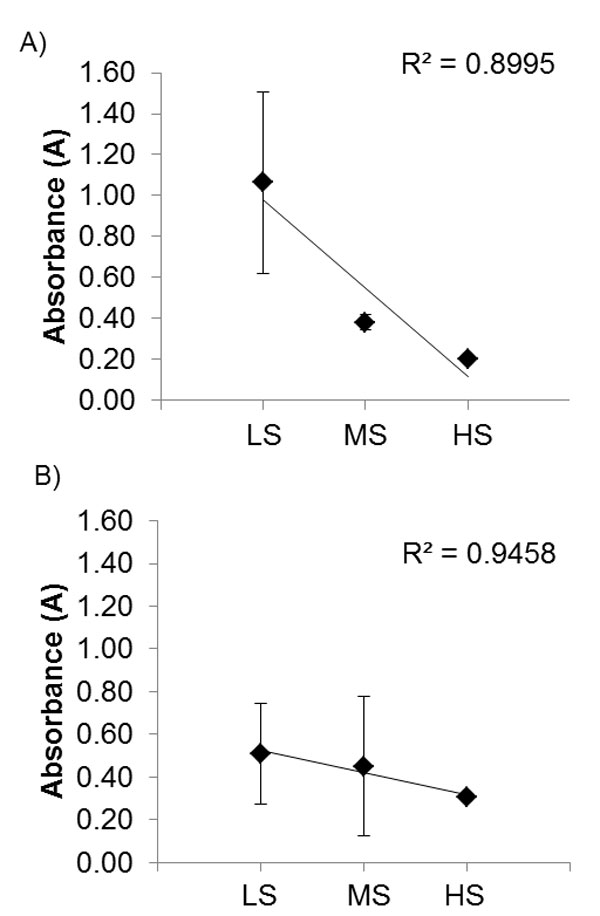
**Average absorbance ± standard deviation of (A) lipid to collagen and (B) calcification to collagen ratio for each plaque group as classified by mechanical stiffness (LS = low stiffness, MS = medium stiffness and HS = high stiffness)**.

Global FTIR spectroscopy analysis identified the functional groups associated with the three key plaque components collagen, lipid and calcification. The absorbance spectral peak-area readings are directly related to the concentration of the components. Figure 3 shows the average absorbance (± standard deviation) values of lipid:collagen and calcification:collagen ratios for each plaque grouping as classified by initial stiffness. Results show that lipid concentrations decrease with increasing mechanical stiffness level. A slight decrease in the calcification concentration is also exhibited which demonstrates no clear correlation with the increasing mechanical stiffness level.

**Figure 4:**
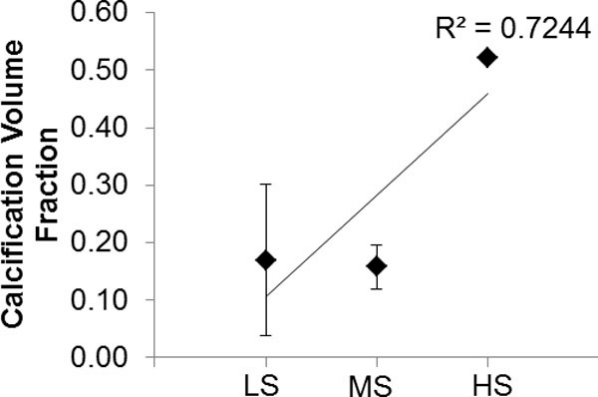
**The average calcification volume fraction ± standard deviation for each plaque group as classified by mechanical stiffness (LS = low stiffness, MS = medium stiffness and HS = high stiffness)**.

µX-CT imaging revealed the calcification present throughout the internal plaque morphology and facilitated the characterisation of calcification using volume and surface area measurements thus complementing the global FTIR biochemical readings. Figure 4 shows the averaged calcification volume fraction measurements (± standard deviation) for each stiffness group. This measure demonstrates that there was a slight change between low and medium stiffness groupings with the exception of one plaque in the lowest stiffness group which had the second highest overall calcification volume faction value of 0.32. A significant increase was found between medium and the high stiffness groupings.

**Figure 5:**
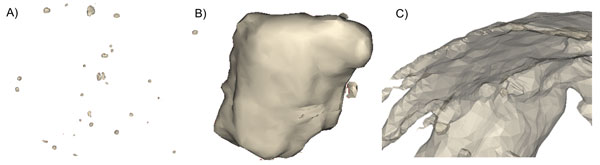
**µX-CT images of calcification structures (A) spherical inclusions (<300 µm), (B) irregular agglomerated macro-nodes and (C) thin sheet-like structures**.

Figure 5 shows an example of the three types of calcification geometries that were identified within the internal morphology of the plaque sections for this sample set. Table 2 summarises the number of each type that was found in the plaque sections. A total of 505 spherical calcification particles were identified in the six plaque sections. A higher concentration of these randomly distributed spherical particles was found in the least stiff, high lipid, plaque type along with thin sheet-like structures. Conversely, the stiffer plaques contained larger irregularly shaped agglomerated nodes of calcification with few spherical nodes. The plaque with the highest stiffness contained a single agglomerated mass of calcification that was the same length as the plaque and was located circumferentially in the cross-section; this plaque also exhibited 113 spherical particles.

**Table 2:** µX-CT classification of calcification volume fraction (CVF) and quantity of individual inclusion type spherical particle, sheet-like and irregular node, for each plaque section examined in this study.

Sample	CVF	Particle	Sheet	Irregular
**LS**	0.11	158	1	12
**LS**	0.32	88	2	1
**LS**	0.08	106	3	7
**MS**	0.19	23	1	1
**MS**	0.13	17	-	4
**HS**	0.52	113	-	1

### Validation

**Figure 6:**
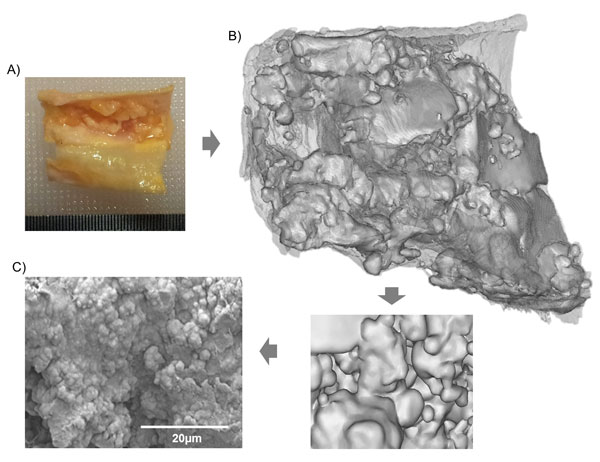
**Characteristic imaging process of (A) advanced carotid plaque with (B) surface defects identified by μX-CT and (C) the surface topology further examined using SEM-EDX chemical analysis highlighting the irregular surface defects on the plaques luminal surface**.

SEM-EDX chemical analysis confirmed the presence of calcification. The presence of calcification was confirmed through the measurement of stoichiometric hydroxyapatite ratio of calcium to phosphorus of 1.67:1 [12]. All regions analysed had an equivalent ratio or greater. Furthermore, the structural plaque characterisation using μX-CT identified regions of interest that warranted further investigation by SEM imaging analysis revealing the surface topography of the plaques. Figure 6 examines the intimal surface irregularity of a severely calcified plaque. According to [12], these carotid plaque surface irregularities are a high risk factor for cerebrovascular events during the endovascular treatments. This intimal surface characteristic was covered with irregular bubbly-like micro nodules.

## Discussion

This study investigates the use of global FTIR and µX-CT as complementary characterisation techniques in order to better understand the mechanical behaviour of diseased plaque tissue. The biochemical analysis identifies the key components in each plaque specimen while the complementary µX-CT imaging modality permits accurate reconstructions of the internal morphology of the carotid plaque specimen's post-mechanical loading. The use of volumetric and surface area measurements of the individual calcification inclusions allowed for a quantitative method of examining the structures. The findings presented in this study suggest that experimentally derived mechanical behaviour and rupture data may be related to the presence of individual calcification geometries.

The juxtaposition of the key pathological components, lipid and calcification identified by global FTIR, embedded in the plaque tissue were assessed individually in order to demonstrate how each component contributes to the structural integrity of the macroscopic plaque structure (Figure 3). The global FTIR analysis shows clear markers for the level of disease progression through the decreasing lipid concentrations as the plaques stiffness increases. This decreasing lipid measure agrees with the AHA plaque classification standard which states that pathologically advanced plaques have lower lipid and are more fibrotic [13]. It has been hypothesised that higher lipid measures also impose an element of vulnerability affecting strength [14]. A similar decreasing trend is observed in the calcification:collagen ratio where calcification concentrations progressively decrease with each increasing stiffness group which suggest that calcification is not contributing to the mechanical stiffness of the plaque despite other findings suggesting that calcification is a marked pathological component that strongly contributes to the mechanical stiffness of a plaque [15]. This lack of correlation between mechanical stiffness may be explained by the finite penetration depth of the global FTIR spectroscope in reaching the outer plaque regions in thicker specimen. Histomorphological studies have demonstrated that calcified tissue can be heavily concentrated in the outer thickness of the atherosclerotic plaque [16].

µX-CT was employed as an additional technique that can identify the entire internal plaque volume therefore providing a global visual representation of the structural inclusions. This was performed in order to address the limited penetration depth of the global FTIR analysis. The µX-CT results of this study shows that applying the overall calcification volume fraction measurements to the mechanical data (Figure 4) arrives at a clearer explanation for the observed increasing initial stiffness levels. The results for this sample set suggest that there may be a threshold level where calcification does not contribute significantly to the mechanical stiffness as there is little variation in the degree of calcification between the low and medium stiffness groups in comparison with the highest stiffness group; that has a calcification volume fraction of 0.52. Further analysis must be carried out to determine how calcification is affecting the varying stiffness levels in a larger sample set. From a clinical perspective, a systematic review that compares the different methods used to quantify calcification has shown that the lower overall calcification measures are more likely to be associated with clinically symptomatic plaques [17]. However, the mechanical behaviour of the plaque under the influence of calcification is not fully understood using overall volume measurements. Alternative methods of measuring calcification are required to better understand the effect that calcification has on mechanical behaviour.

In this study the individual inclusions were analysed in order to assess if specific geometries contribute to the experimentally derived mechanical properties. In this sample set, the three distinct calcification geometries identified were micro spherical inclusions (<300 µm), long thin sheet-like structures and irregularly shaped agglomerated macro-nodes (Figure 5).

Computational studies have predicted that micro spherical calcifications can cause localised stress concentrations in the tissue structure inducing a weakening effect that can diminish the ultimate strength [18]. In this study, higher quantities of smaller micron scale spherical particles were found in the least stiff group with the weakest ultimate strength response (Figure 2). These spherical inclusions can be a potentially dangerous type of calcification depending on location where they have been linked to the cause of fatigued fibrous cap rupture [19].

In contrast to these findings, Buffington et al. have recently highlighted the importance of macro-calcification analysis and have predicted that an increase in localised stress concentrations can be dependent on the geometrical parameters including shape, size and location of the macro inclusions [20]. The sheet-like and irregularly shaped agglomerated macro geometries identified in this study have been previously identified through the use of *in vitro *SEM analysis of cross-sections of plaque [5,21,22]. Similar calcification geometries have also been documented in a clinical study that correlates calcification geometries and culprit lesions. Spotty patterns of calcification were typically associated with patients with acute myocardial infarction whereas patients with stable angina pectoris were associated with longer more extensive calcifications [23].

In this sample set it would appear that sheet-like structures are associated with the lower stiffness plaque type. Sheet-like structures that lie in the circumferential thickness have been linked to the failure of clinical procedures [3,24].The results also show that the irregularly shaped agglomerated macro-nodes are a common geometry in the more advanced stiffer carotid plaques. These irregular nodes are regarded as a calcification type commonly associated with the carotid artery vasculature [25]. The most severely stiffened mechanical response was as a result of the presence of a single mass of calcification which dominated the length of the plaque and was located circumferentially in the plaques cross section. This structure appears to have formed from agglomerated nodes. *In vivo*, similar circumferential calcified plaques have been shown to experience post-stenting geometrical alterations [26]. Post-stenting analyses, carried out by multi-detector computed tomography (MDCT), show how calcification fragmentations may occur where the calcification divides into several pieces of various sizes while other calcification experience cracking without fragmentation [27].

This study demonstrates a characteristic process of multitudinous diagnostic capability that is required in order to solve the multifactorial biomechanics problem. FTIR analysis is a capable technique for examining the specific plaque components through the use of a catheter based device [28] while CT imaging is available in a clinical setting. The conflicting methods of calcification measurements require further investigation. Future studies should also incorporate the density of the calcification as a parameter of importance as recent studies have shown an inverse relationship between cardiovascular risk and calcification density [29]. Furthermore, as trends are shifting toward the use of calcification as a measure for plaque characterisation, standardised methods are needed to lead to a better informed method of predicting plaque suitability for minimally invasive intervention [30]. Plaque assessment via imaging modalities need to include calcification geometrical features, shape, size and location in order to accurately evaluate the plaque in question.

A limitation of this study is the examination of a small sample size of six human carotid plaques, which limits the conclusions that can be drawn regarding the mechanical behaviour. However, it should be noted that this initial investigation was primarily concerned with assessing the feasibility of improving the predictive value of using calcification to understand the mechanical properties of plaque. A limitation regarding the structural characterisation is that it requires a tissue preservation process to prevent tissue degradation during the long scan times and for protection from intense x-ray source in the SEM which can result in tissue damage. Shorter scan times may eliminate the need for tissue preservation along with the use of a low vacuum SEM which requires little sample preparation. Uniaxial *in vitro *testing does not fully describe the behaviour of a plaque *in vivo *as it only incorporates one aspect of the physiological loading. *In vitro *experimental testing of carotid plaque specimens offers a method of ascertaining biomechanical parameters, rupture behaviour and mechanical response to large deformation in the circumferential loading direction, which cannot be examined through *in vivo *material characterisation. The experimental testing in this study employs the standardised test method for uniaxial testing of diseased arterial tissue in order to allow for a better comparison with other *in vitro *studies [31]. μX-CT scanning could not differentiate the lipid component from the plaque tissue as a result of poor contrast differentiation. It has been suggested that the use of osmium tetroxide in tissue preparation allows the identification of the lipid content [32]. However, it leads to arbitrary segmentation and a degree of overlap between the similar tissue densities and therefore was not implemented for this study. Furthermore, spectroscopic FTIR analysis adequately identified the lipid composition in the plaque specimen.

## Conclusion

This study demonstrates a method of characterising human atherosclerotic carotid plaque tissue's composition and morphology using combined spectroscopic and imaging modalities. The complementary characterisation shows that the presence of calcification strongly influences the heterogeneous mechanical behaviour of the carotid plaque specimens in response to circumferential loading. The overall calcification volume fraction measurement is an effective marker to predict the mechanical stiffness behaviour of the plaque, where increasing measures relate to increased stiffness. The findings in this study warrant further investigation in a large cohort of plaques in order to examine the dominating calcification geometries and associated mechanical material plaque properties. Also, the identification of the particular calcification features and their influence on mechanical properties may help to clarify the mechanism of stent expansion in calcified carotid plaque lesions and elucidate the procedural risk of endovascular treatments in plaque with certain calcification types.

## List of abbreviations

FTIR: Fourier Transform Infrared; ATR: Attenuated Total Reflectance; SEM: Scanning Electron Microscopy; EDX: Energy Dispersive X-ray; CVF: Calcification volume fraction; μX-CT: Micro x-ray computed tomography; Lip: Lipid; Col: Collagen; Calc: Calcification;

## Competing interests

The authors declare that they have no competing interests.

## Ethics and consent

Carotid plaque specimens were acquired from standard endarterectomy procedures performed in the University of Limerick Hospital Ireland in a manner that conformed to the Declaration of Helsinki and was approved by the hospital's Ethical Research Committee.

## Authors' contributions

HB was the lead researcher of this paper and MW supervised all work performed. HB ascertained the micro x-ray tomography (μX-CT) data for this study and subsequently interpreted the data in order to develop a correlative approach using the FTIR and μX-CT characterisation techniques. JM initially developed a FTIR classification method. HB developed a new classification method based on a subset of the samples that were mechanically tested and characterised by JM in order to test the feasibility of the proposed methodology. HB performed Scanning Electron Microscopy-Energy Dispersive X-ray Analysis (SEM-EDX) analysis to validate the μX-CT data. HB, EC and MW were all involved in the conception and design of the article as well as the drafting of the manuscript. HB, JM and EC all contributed to the initial development of the discussion points outlined in this study. HB, EC and MW critically reviewed the submitted manuscript to ensure accuracy of the data and its interpretation. HB, EC, JM, and MW are all accountable for every aspect of the work and ensure that the data and points made in this manuscript are accurate and are completely factual based.

## Declarations

Publication of this article was paid with funding from Irish Research Council Grant no. (GOIPG/2013/1309).

This article has been published as part of *BioMedical Engineering OnLine *Volume 14 Supplement 1, 2015: Cardiovascular Disease and Vulnerable Plaque Biomechanics. The full contents of the supplement are available online at http://www.biomedical-engineering-online.com/supplements/14/S1
